# Racial and Ethnic Data Reported for Peanut Allergy Epidemiology Do Little to Advance Its Cause, Treatment, or Prevention

**DOI:** 10.3389/fpubh.2021.685240

**Published:** 2021-10-27

**Authors:** Nigel Mark Thomas

**Affiliations:** Bronx Community College, The City University of New York, New York, NY, United States

**Keywords:** peanut allergy epidemiology, Hispanic American adults, African American adults, household income, Asian American adults

Although allergies to peanut legumes affect less than two percent (1.8%) of the United States population (1), it is not an inconsequential public health concern. For persons allergic to the legume, its ingestion triggers harmful immune responses. The most severe response, anaphylaxis, can cause symptoms of swelling, hives, throat tightening, breathing difficulties, lowered blood pressure, and shock. An estimated 40% of children with a food-related allergy have experienced at least one anaphylactic event (2). Estimates are that about 13 people die from peanut-related anaphylaxis each year (3). For those living with peanut allergies their health-related quality of life (HRQoL) i.e., their ability to live fulfilling lives is impacted. When compared with the population without peanut allergies there are more emergency room (ER) visits, greater levels of anxiety among caregivers as well as career and work disruptions. Among children with peanut allergies one half have had one or more lifetime emergency room visits, and one in five have had an ER visit in the past 12 months (4). For both children and adults with peanut allergies the experience presents considerable levels of stress, anxiety, and uncertainty. About four out of five adolescents anticipated and feared negative events and this affected their emotional well-being “somewhat.” For their overall quality of life, 26.5% of adolescents, 31.4% of adult patients, and 34.3% of caregivers indicated the peanut allergy's intrusion to be “very much” or “completely.” About five percent of caregivers reported quitting a job because of their child's condition, 2.5% had to change jobs, and 1.9% lost their jobs (5, 6). The prevalence of peanut allergies has increased in the last two decades. With most prior studies focused on the experience of children, recent research that delineates adult-onset peanut allergies is welcome, however, the non-medical data collected are unlikely to increase our understanding of its etiology, inform evidence-based public health interventions, or redress health inequities.

The representative, probability-based sample of U.S households tells us that income, ethnicity, and race are risk factors for adult-onset peanut allergy.

Findings are that individuals living in households earning between $50,000 and $149,999 (1) are more likely to report peanut allergies than those from households in different income tiers. Households with annual incomes between $50,000 and $99,999 account for 36.8% of those with peanut allergies but 30.8% of the U.S. population. Households with annual incomes between $100,000 and $149,999 account for 22.2% of those with peanut allergies but 19.6% of the U.S. population. Hypotheses have long abounded about a positive relationship between income and food allergies. One theory is that higher incomes mean more education. More education means greater awareness about peanut allergies. Greater awareness becomes a precursor to avoidance. Avoidance paradoxically increases hypersensitivity. A different theory suggests that higher incomes are associated with altered diets, more antibiotic use, and less complex environmental exposures. In tandem, these factors result in less diverse human microbiota. Less diverse microbiota presage immuno-intolerance and increased risk of allergies. To say that a person has 1.2 greater odds of acquiring a peanut allergy if the annual household income is $50–$99,999 is akin to a modern-day etiological myth. It is unlikely that an individual's transition to a different household income tier remedies the peanut allergies. And, what is one to make of the finding that living in a household where annual income exceeds $150,000 appears to be protective and so diminishes a person's susceptibility to the peanut allergy? More than a century ago Dr. Herbert West reminded us that “correlation does not imply causation” and the adage holds true today.

To untangle the income-peanut allergy connection would require inter-generational studies of geography, psychology, and physiology. More promising data collection than income that might inform public health interventions include dietary changes, volume of diverse microbiota, and environmental exposures.

The data collection and reporting of ethnicity as a variable in peanut allergy prevalence might be even more spurious than income. Hispanic adults are over-represented among those with peanut allergies. They account for 20.9% of those with peanut allergies, but 15.4% of the U.S. population (1). It is unlikely that Hispanics have genetic predispositions to peanut allergies because available data indicate lower prevalence in many Latin American countries compared to the United States. Research carried out in Mexico, for instance, finds lower levels of peanut sensitization compared to the United States (7). There are at least 25 countries included in the United States “Hispanic” taxonomy; the classification masks crater-like chasms in health beliefs, healthcare access, dietary practices, and health outcomes among different sub-populations.

Similar concerns exist for data about Asian Americans. They account for 6.3% of those with peanut allergies, but 3.8% of the U.S. population (1). Peanut allergies are less prevalent in many Asian countries compared to the United States. Epidemiological investigations among children and adults in Singapore, for example, confirmed the rarity of severe peanut allergies resulting in anaphylaxis. The researchers documented only two cases among adults and none among children. An anaphylaxis study from neighboring Thailand with both adult and child participants documented no cases of peanut allergies. Food preparation can differ significantly across the continent. Whereas, peanuts figure so prominently in food preparation in Myanmar and Cambodia that some residents cannot fathom the concept of peanut allergy, peanuts are uncommon in food preparation in Japan and India (8). In the context of the United States the umbrella term “Asian American” encapsulates dozens of countries with diverse health beliefs, healthcare access, dietary practices, and health outcomes.

There are three theories that may explain why some ethnicities are at higher risk of peanut allergenicity: migrant selectivity; epigenetics; and Western adaptation. Migrant selectivity tells us that migrants are not necessary representative of their origin countries so that their risk may be dissimilar from compatriots. Epigenetics acknowledges that heritable changes can be caused by the activation and deactivation of genes without any change in the underlying DNA sequence of the organism. Peanut allergies could be influenced by genes interacting in complex ways with the environment. Some ethnicities may have genetic predisposition to peanut allergies and the new environment no longer shields them from that propensity. The Western adaptation hypothesis makes the case that how peanuts are prepared in origin and host countries makes a difference. In China, peanuts are usually fried or boiled, while in the U.S. they are more commonly dry roasted (9). Different food preparation methods among different ethnicities could be linked to risk differentials. More promising data collection than ethnicity that might inform public health interventions include country of origin, acculturation, food preparation, and dietary changes, volume of diverse microbiota, and environmental exposures.

Non-Hispanic Blacks are also over-represented among adults with peanut allergies. They account for 15.5% of those with peanut allergies, but 11.6% of the U.S. population (1). Even though genetically verified African ancestry has been linked to peanut allergies (10), a caveat is that other studies report different sub-populations in a single country with differential risks for peanut allergies (11). If the results for South Africa are replicated across the more than fifty countries on the continent and, then, factored with the genetic inheritance of non-Hispanic Blacks, the story is no longer so straightforward. A compelling argument for there being an environmental role is that many non-Hispanic Blacks are thought to have ancestry in West Africa. In the two largest West African countries by population, Nigeria and Ghana, peanuts are main ingredients in stews and marinades, but severe allergic reactions are rare.

This author performed a database search In February 2021 with the search terms, “peanut allergy,” “United States.” That search turned up 21,400 results. A separate search with the words “peanut allergy,” “United States,” “statistical survey” yielded 17,200 results. This, of course, is not meant as a validated method of how popular statistical surveys are in peanut allergy epidemiological studies but taken, in context, with studies such as one the reported here, they do provide clues about recent trends and directions in scholarship.

Statistical surveys are beneficial because they can be developed more quickly and more cost-effective than other data-collection methods, and they provide information about “representative” national samples. Surveys are only as valuable as the questions they ask, though. Recent survey questions implicating income, ethnicity, and race as risk factors for peanut allergies do not advance our understanding of etiology or public health intervention and may even be harmful for addressing social determinants of health and achieving health equity.

For sidestepping the etiological myths, we need “life course” research that includes variables measuring gestation, country of origin, age introduced to peanuts, parental histories with peanuts, places people have lived, social status, acculturation, race, microbiota, genes, dietary habits, adverse childhood experiences, adverse adolescent experiences, adverse young adulthood experiences, and adverse midlife experiences. The peanut allergy epidemiolocal scholarship to-date makes a convincing case that the complex and intertwined biological, behavioral and psychosocial variables that operate across the life span are the most promising approaches to understanding its etiology rather than a focus on independent variables of income, race, and ethnicity (12). Ironically, the proposed research designs will better equip us to understand the roles of social determinants including income, ethnicity, and race so we can create effective strategies for health equity.

In the absence of peanut allergy causal and contextual factors that are malleable, the priority for public health outreach should be on education about prevention and management. Every child, adult, and caregiver benefits from knowing The National Institute for Allergy and Infectious Disease (NIAID) (13, 14) recommended ages to introduce peanuts to children. A low-risk child has no eczema or food allergies and can be introduced to peanut-based foods consistent with family practices. The moderate-risk group includes children who have mild-to-moderate eczema, or there are no already present food allergies (such as an allergy to eggs). The guidelines recommend parents feed peanut-based foods at around 6 months of age. The highest risk group includes children with moderate-to-severe eczema and/or an egg allergy or children with an immediate relative who is also allergic. An allergist should evaluate infants in the high-risk category before the introduction of peanut-based foods. The earliest age of introduction for high-risk children is between 4 ad 6 months of age.

Every child, adult, and caregiver benefits from knowing the symptoms of anaphylaxis. Individuals with peanut allergies are always recommended to carry two epinephrine auto-injectable devices with them along with an Allergy and Anaphylaxis Emergency Plan.

For the ~7%, (5) of people with peanut allergies and dissatisfied with current approaches to avoid or prevent reactions, there should be meaningful dialogue between allergist and patient/caregiver about alternatives, risks, and benefits of FDA approved treatments.

## Author Contributions

The author confirms being the sole contributor of this work and has approved it for publication.

## Conflict of Interest

The author declares that the research was conducted in the absence of any commercial or financial relationships that could be construed as a potential conflict of interest.

## Publisher's Note

All claims expressed in this article are solely those of the authors and do not necessarily represent those of their affiliated organizations, or those of the publisher, the editors and the reviewers. Any product that may be evaluated in this article, or claim that may be made by its manufacturer, is not guaranteed or endorsed by the publisher.
