# Supplementary material for: Racial and Ethnic Data Reported for Peanut Allergy Epidemiology Do Little to Advance Its Cause, Treatment, or Prevention
Source: Front Public Health. 2021 Oct 27;9:685240. doi: 10.3389/fpubh.2021.685240 (PMC8578288; doi:10.3389/fpubh.2021.685240)

Race/Ethnicity	United States' Adults without Current Peanut Allergies	United States' Adults with Current Peanut Allergies
Asian (non-Hispanic)	3.8%	6.3%
Black (non-Hispanic)	11.6%	15.5%
Hispanic	15.4%	20.9%
Other	69.2%	57.3%

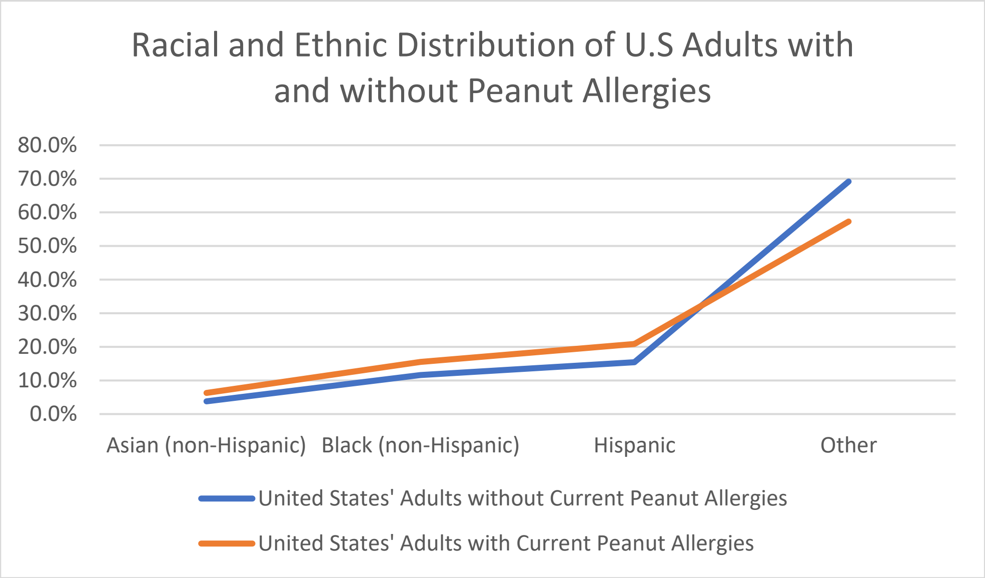

Supplement: Supplementary file 1 [file Data_Sheet_1.PDF]
